# A phase II clinical trial of frameless, fractionated stereotactic radiation therapy for brain metastases

**DOI:** 10.1093/jncics/pkad093

**Published:** 2023-11-07

**Authors:** Amit K Garg, Mike Hernandez, Pamela J Schlembach, John R Bowers, Mary F McAleer, Paul D Brown, Ramesh Gopal, Lee Wiederhold, Todd Swanson, Shalin J Shah, Jing Li, Sherise D Ferguson, Nancy V Philip, Lilybeth DeGracia, Elizabeth S Bloom, Stephen G Chun

**Affiliations:** Department of Radiation Oncology, Presbyterian Healthcare Services, Albuquerque, NM, USA; Department of Biostatistics, The University of Texas MD Anderson Cancer Center, Houston, TX, USA; Department of Radiation Oncology, The University of Texas MD Anderson Cancer Center, Houston, TX, USA; Department of Radiation Oncology, Presbyterian Healthcare Services, Albuquerque, NM, USA; Department of Radiation Oncology, The University of Texas MD Anderson Cancer Center, Houston, TX, USA; Department of Radiation Oncology, Mayo Clinic, Rochester, MN, USA; Department of Radiation Oncology, University of New Mexico, Albuquerque, NM, USA; Department of Radiation Oncology, The University of Texas MD Anderson Cancer Center, Houston, TX, USA; Department of Radiation Oncology, The University of Texas MD Anderson Cancer Center, Houston, TX, USA; Department of Radiation Oncology, The University of Texas MD Anderson Cancer Center, Houston, TX, USA; Department of Radiation Oncology, The University of Texas MD Anderson Cancer Center, Houston, TX, USA; Department of Neurosurgery, The University of Texas MD Anderson Cancer Center, Houston, TX, USA; Department of Radiation Oncology, The University of Texas MD Anderson Cancer Center, Houston, TX, USA; Department of Radiation Oncology, The University of Texas MD Anderson Cancer Center, Houston, TX, USA; Department of Radiation Oncology, The University of Texas MD Anderson Cancer Center, Houston, TX, USA; Department of Radiation Oncology, The University of Texas MD Anderson Cancer Center, Houston, TX, USA

## Abstract

Stereotactic radiation therapy yields high rates of local control for brain metastases, but patients in rural or suburban areas face geographic and socioeconomic barriers to its access. We conducted a phase II clinical trial of frameless, fractionated stereotactic radiation therapy for brain metastases in an integrated academic satellite network for patients 18 years of age or older with 4 or fewer brain metastases. Dose was based on gross tumor volume: less than 3.0 cm, 27 Gy in 3 fractions and 3.0 to 3.9 cm, 30 Gy in 5 fractions. Median follow-up was 10 months for 73 evaluable patients, with a median age of 68 years. Median intracranial progression-free survival was 7.1 months (95% confidence interval = 5.3 to not reached), and median survival was 7.2 months (95% confidence interval = 5.4 to not reached); there were no serious adverse events. Outcomes of this trial compare favorably with contemporary trials, and this treatment strategy provides opportunities to expand stereotactic radiation therapy access to underserved populations.

Brain metastases are a common manifestation of advanced cancer, seen in more than 100 000 patients in the United States annually (1). For low-volume brain metastases, stereotactic radiation therapy (RT) has emerged as the standard of care to attain local control while mitigating the risk of radiation-induced neurocognitive toxicity (2-5). Although advanced radiosurgery platforms are now widely available at many community and academic radiation clinics in urban centers, there can be substantial barriers to access for those in suburban, rural, or minority communities (6). In part to improve access to stereotactic RT, frameless, fractionated stereotactic RT series for brain metastases have increasingly been reported (7-9). To validate the efficacy of a 3 to 5 fraction frameless, fractionated stereotactic RT regimen for intact brain metastases from solid malignancies, we conducted a pragmatic, prospective phase II clinical trial of frameless, fractionated stereotactic RT for intraparenchymal brain metastases in an integrated academic satellite network for patients unwilling or unable to travel to the main academic campus for stereotactic RT with a frame.

Institutional review board approval was obtained for this prospective, multi-institutional, prospective phase II clinical trial, as shown in Figure 1 (2015-0874). The trial was registered at ClinicalTrials.gov under identifier NCT02798029. Written informed consent was obtained from all participants. Patients 18 years of age or older with 4 or fewer intraparenchymal brain metastases 5 cm or smaller in their largest dimension from a known, biopsy-proven cancer who were unable or unwilling to travel to the main campus for radiosurgery with a frame were eligible. Ineligibility criteria included metastases involving the brainstem or optic pathway, inability to tolerate a magnetic resonance imaging scan, pregnancy, histology unsuitable for stereotactic RT (small cell lung cancer, myeloma, lymphoma and leukemia), or history of whole-brain RT. The primary endpoint of this trial was to assess efficacy of frameless, fractionated stereotactic RT, defined as local control of the treated index lesion at 6 months. Secondary endpoints included time to local failure, intracranial progression-free survival (PFS), overall survival, and Common Terminology Criteria for Adverse Events (CTCAE), version 4, toxicities. Patients were immobilized using a standard thermoplastic mask manufactured by Orfit Industries (Wijnegem, Belgium) and bite block for computed tomography simulation and treatment. An intravenous contrast–enhanced, 1-mm, sliced computed tomography scan was used for planning fused with a volumetric magnetic resonance imaging scan of the brain obtained within 2 weeks of treatment to delineate the gross tumor volume. The planning target volume was developed through a 2-mm geometric margin on gross tumor volume. Radiation dose was prescribed to provide more than 95% planning target volume prescription dose coverage and a conformity index (volume of prescription dose / volume of planning target volume) less than 1.5 using volumetric arc therapy. The dose prescriptions to the planning target volume were determined based on maximum diameter of the index brain lesion (gross tumor volume): less than 3.0 cm, 27 Gy in 3 fractions and 3.0 to 3.9 cm, 30 Gy in 5 fractions. Radiation plans were optimized based on normal tissue constraints provided in Supplementary Table 3 (available online). Radiation plans were subject to prospective quality assurance review before delivery. For daily frameless, fractionated stereotactic RT delivery, a cone beam computed tomography scan was used for image guidance, and the image was verified by the treating physician before each treatment. Patients were followed up with a gadolinium contrast–enhanced magnetic resonance imaging scan of the brain and clinically evaluated every 3 months for 1 year. Toxicity was assessed using CTCAE, version 4. Neurologic toxicity monitoring was conducted independently for each study cohort using the method described by Thall et al. (10), where the rate of CTCAE grade 3 or higher treatment-related neurologic toxicity from first frameless, fractionated stereotactic RT treatment to 3-month follow-up was sequentially monitored. The stopping rules included the determination of more than a 90% likelihood of a CTCAE grade 3 or higher neurologic toxicity rate greater than 33% at any point in the trial or evidence of futility, defined as a 6-month local control rate of 55% or lower. Descriptive statistics were used to describe patient characteristics and study data. Intracranial PFS (defined as time from enrollment to either first progression in the brain or death from any cause) and overall survival were evaluated using the Kaplan-Meier method. For intracranial PFS, patients not experiencing death or intracranial progression were censored at the last protocol-mandated tumor assessment. Local failure was defined as at least a 20% increase in the sum longest distance relative to lesion size before RT and was estimated at each follow-up as previously described (11). We hypothesized a 6-month local control rate of 75% or more and tested this hypothesis using a 1-sided test (α = .05, β = .2), with a null hypothesis of a local control rate of 55% or more at 6 months. The probability of stopping was .409 for the null hypothesis and .014 for the alternate hypothesis. A 2-sided exact 95% confidence interval was used to further characterize local control at the lesion level. Statistical analyses were conducted using R, version 4.1.2 (R Foundation for Statistical Computing, Vienna, Austria) and Stata, version 16 (StataCorp, LLC, College Station, TX). The data underlying this article are available in this article and its Supplementary Material (available online).

**Figure 1.**
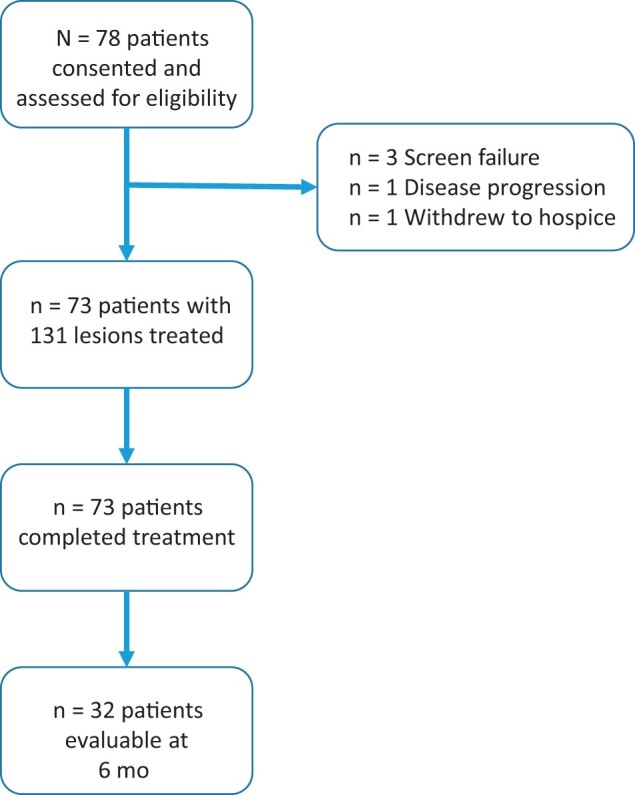
Frameless, fractionated stereotactic radiation therapy phase II clinical trial profile.

A total of 78 patients consented to participate in the trial, of whom 1 patient withdrew, 1 patient was removed due to disease progression, and 3 patients were removed who did not meet entry criteria (Figure 1). The baseline characteristics of the 73 evaluable patients are shown in Supplementary Table 1 (available online). Median participant age was 68 years, 58% of the patients were female, 42% were male, 2.7% were American Indian and/or Alaskan Native, 1.4% were Asian, 2.7% were Black and/or African American, and 82% were White. Eastern Cooperative Oncology Group (ECOG) performance status ranged from 0 to 4, and 89% of patients had and ECOG performance status of 2 or less. The most common histology was non–small cell lung cancer (51%), followed by breast cancer (14%). There were 127 lesions measuring less than 3.0 cm treated with 27 Gy in 3 fractions and 4 lesions measuring 3.0 to 3.9 cm treated with 30 Gy in 5 fractions. Although potentially eligible, no patients who enrolled had metastases 4 to 5 cm in size. For evaluable patients, median follow-up was 10 months, and oncologic outcomes were characterized by Kaplan-Meier method (Figure 2). The local control rate of treated index lesions were 93.2% at 6 months and 91.8% at 12 months (Figure 2, A). Median intracranial PFS was 7.0 months (95% confidence interval = 5.26 months to not reached), as shown in Figure 2, B. Median survival (Figure 2, C) was 7.2 months (95% confidence interval = 5.42 to not reached). Protocol-related CTCAE adverse events are shown in Supplementary Table 2 (available online). No protocol-related CTCAE grade 3 or greater adverse events or symptomatic radiation necrosis was observed.

**Figure 2.**
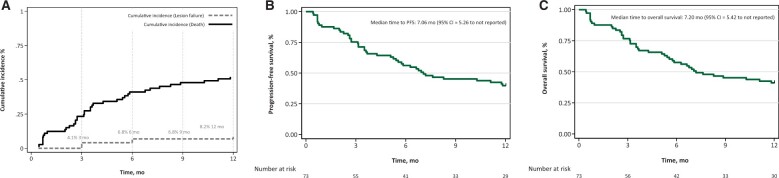
**A)** Cumulative incidence of local lesion failure, with death as a competing risk. **B)** Kaplan-Meier plot of intracranial progression-free survival (PFS). **C)** Kaplan-Meier plot of overall survival.

In summary, we conducted a pragmatic phase II clinical trial evaluating 3- to 5-fraction frameless, fractionated stereotactic RT for intraparenchymal brain metastases in an integrated academic satellite network for patients in suburban and rural communities. This frameless, fractionated stereotactic RT regimen succeeded in achieving the hypothesis pertaining to the primary trial endpoint. It is notable that both the local control and overall survival rates observed in this trial compare favorably to contemporary clinical trials for this patient population (2,5,8,12-14). Based on these results, this frameless, fractionated stereotactic RT regimen warrants further consideration and investigation, particularly for patients who cannot access more specialized stereotactic RT platforms. This frameless, fractionated stereotactic RT regimen may also have a role in future prospective brain metastasis trials to increase patient participation. Future study is planned to examine the cost-effectiveness and other factors affecting long-term outcomes for this treatment regimen.

## Supplementary Material

pkad093_Supplementary_Data

## Data Availability

The data underlying this article are available in this article and its online supplementary material.
