# Supplementary material for: A phase II clinical trial of frameless, fractionated stereotactic radiation therapy for brain metastases
Source: JNCI Cancer Spectr. 2023 Nov 7;7(6):pkad093. doi: 10.1093/jncics/pkad093 (PMC10715838; doi:10.1093/jncics/pkad093)
Supplement: pkad093_Supplementary_Data [file pkad093_supplementary_data.zip › JNCI Brain Metastasis supplemental appendix 11_03_2023.pdf]

Supplementary Materials

Table 1. Baseline patient characteristics.

	N=73
**Age (years)**	68, (range 42-90)
**Gender**	
Male	31 (42%)
Female	42 (58%)
**Race**	
American Indian/Alaskan Native	2 (2.7%)
Asian	1 (1.4%)
Black/African American	2 (2.7%)
Pacific Islander	1 (1.4%)
Unknown	7 (9.5%)
White/Caucasian	60 (82.2%)
**Eastern Cooperative Group (ECOG) Performance Status**	
0	9 (12%)
1	42 (58%)
2	14 (19%)
3	7 (9.6%)
4	1 (1.4%)
**Primary Tumor Origin**	
Bladder	1 (1.4%)
Breast	10 (14%)
Colorectal	3 (4.1%)
Gastroesophageal	3 (4.1%)
Gynecologic	3 (4.1%)
Head & neck	2 (2.7%)
Renal	4 (5.5%)
Non-small cell lung cancer	37 (51%)
Sarcoma	3 (4.1%)
Melanoma	3 (4.1%)
Skin (non-melanoma)	3 (4.1%)
Unknown primary	1 (1.4%)

Table 2. Protocol-related Common Terminology Criteria for Adverse Events version 4 toxicities.

Grade 1		Grade 2	
Alopecia	N = 18	Alopecia	N = 3
Dizziness	N = 1	Fatigue	N = 4
Fatigue	N = 4	Headache	N = 1
Headache	N = 2	Nausea	N = 1
Nausea	N = 1		

Table 3. Protocol FFSRT dose constraints. Dmax, maximum dose; cc, cubic centimeter.

Organ at Risk	27 Gy in 3 fractions<br>(GTV < 3 cm)	30 Gy in 5 fraction<br>(GTV 3.0-4.0 cm)
Brainstem	Dmax < 16 Gy, 1 cc < 12 Gy	Dmax < 22 Gy, 1 cc < 20 Gy
Optic Nerves	Dmax < 16 Gy, 1 cc < 12 Gy	Dmax < 22 Gy, 1 cc < 20 Gy
Optic Chiasm	Dmax < 16 Gy, 1 cc < 12 Gy	Dmax < 22 Gy, 1 cc < 20 Gy
Brain	Mean < 8 Gy	Mean < 8 Gy

Study Protocol:
A Phase II Study of the Efficacy, Safety, and Cost of Frameless Fractionated Stereotactic Radiation for Parenchymal Brain Metastases

1.0 Objectives

Primary Objectives

1. To assess the efficacy and safety of frameless fractionated stereotactic radiation therapy (FFSRT) on the treatment of solitary and oligometastatic brain metastases in the MD Anderson Houston Area Locations, MD Anderson Radiation Treatment Centers in New Mexico, MD Anderson affiliates and the main campus of MD Anderson, for patients unable or unwilling to undergo frame-based stereotactic radiosurgery (SRS).

Secondary Objectives

1. To assess 6-month local control, intracranial progression-free survival and overall survival.

2. To collect data on charges and reimbursements of patients treated with FFSRT to compare those charges and reimbursements if the same patients had been treated with single-fraction, frame-based Gamma Knife SRS.

2.0 Rationale

Solitary and oligometastatic brain disease has been effectively managed with frame-based SRS in a single fraction with Gamma Knife, Cyber Knife or linac-based treatment delivery. (1) However, there are published data to suggest that similar local control and toxicity profiles can be achieved with FFSRT. (2,3,4,5) If, in fact, FFSRT is as effective and safe as frame-based single fraction SRS, then more patients would have access to the therapy, since 1) a neurosurgical procedure with frame would not be necessary, and 2) the equipment necessary for FFSRT is more readily available at radiation oncology facilities. In the setting of heath care cost containment, if FFSRT is proven to be as effective as frame-based, single-fraction SRS, it would not be necessary to purchase the additional equipment otherwise needed for centers to offer the frame-based treatment for patients with oligometastatic brain disease. In the setting of the MD Anderson Regional Care Centers, currently only The Woodlands center offers frame-based SRS. Therefore, all patients eligible for focal treatment for their oligometastatic disease must be referred either to the Texas Medical Center or to The Woodlands facility. The additional travel, time and expense may be a burden for patients, who often have limited life expectancy.

3.0 Type of Subjects to be Studied

All patients with 1-4 metastatic brain lesions who are considered eligible for single-fraction, frame-based SRS, who are unable or unwilling to undergo frame-based SRS.

Eligibility

Patients and/or their disease must fulfill the following criteria to be enrolled in this prospective study:

1. Greater than or equal to 18 years of age.

2. One to 4 untreated metastatic brain lesions.
3. Each brain lesion must be less than or equal to 5 cm in diameter and not an optimal surgical candidate.
4. Patient must be able have an MRI of the brain for treatment planning.
5. Histologic confirmation of malignancy.
6. For patients of childbearing potential, non-pregnant state, confirmed by negative serum or urine beta-HCG within (7) days of planned radiation treatment.
7. GFR adequate for IV contrast delivery for imaging.
8. No concurrent chemotherapy.
9. Patient may have had prior therapy for brain metastasis, including radiosurgery and surgical resection at the discretion of the treating physician however only new untreated lesions will be followed on protocol.

Ineligibility

Patients and/or their disease meeting any of the following criteria are ineligible for enrollment.

1. Less than 18 years old.
2. Five or more metastatic brain lesions.
3. Brain lesion(s) greater than 5 cm in diameter.
4. Lesion(s) involving the brainstem, optic chiasm or optic nerve(s).
5. Patients unable to have IV contrast for CT and MRI imaging.
6. Patient unable to have an MRI of the brain.
7. Patients willing to be treated with frame-based Gamma Knife SRS at MD Anderson main campus or MD Anderson at The Woodlands.
8. Positive pregnant status confirmed by serum or urine pregnancy test.
9. Primary small cell lung cancer, myeloma, lymphoma, leukemia, or other histologies not optimally treated with SRS.
10. Patients currently receiving chemotherapy/biologic/immunotherapy as these need to be held during FFSRT
11. Prior whole brain radiotherapy or conventional external beam radiotherapy.

4.0 Research Plan and Methods

The study utilizes a two-stage design with an inherent interim analysis after the first stage to stop the study early for futility. In the first stage of the study, the first 20 evaluable (defined as patients who complete the 6 month follow up) patients (See study design below) will be analyzed prior to making a decision to continue to the second stage of the study. Study data will be collected and managed using REDCap (Research Electronic Data Capture) electronic data capture tools hosted at MD Anderson. REDCap (www.project-redcap.org) is a secure, web-based application with controlled access designed to support data capture for research studies, providing: 1) an intuitive interface for validated data entry; 2) audit trails for tracking data manipulation and export procedures; 3) automated export procedures for seamless downloads to common statistical packages; and 4) procedures for importing data from external sources.[22] In the case of multi-center studies REDCap uses Data Access Groups (DAGs) to ensure that personnel at each institution are blinded to the data from other institutions. REDCap (https://redcap.mdanderson.org) is hosted on a secure server by MD Anderson Cancer Center's Department of Research Information Systems & Technology Services. REDCap has undergone a

Governance Risk & Compliance Assessment (May 2014) by MD Anderson's Information Security Office and found to be compliant with HIPAA, Texas Administrative Codes 202-203, University of Texas Policy 165, federal regulations outlined in 21CFR Part 11, and UTMDACC Institutional Policy #ADM0335.

Those having access to the data include the study PI and research team personnel. Users are authenticated against MDACC's Active Directory system. External collaborators are given access to the database once approved by the PI, with their access expiring in 6 months but renewable in 6 months increments at the request of the PI. The application is accessed through Secure Socket Layer (SSL). All protected health information (PHI) will be removed from the data when it is exported from REDCap for analysis. All dates for a given patient will be shifted by a randomly generated number between 0 and 364, thus preserving the distance between dates. Dates for each patient will be shifted by a different randomly generated number.
Following publication study data will be archived in REDCap. Since study data may be useful for future research studies performed under separate IRB approved protocols, study data will be archived indefinitely in REDCap. Since REDCap is a secure electronic database with controlled access, and because patient identifiers may be needed to link study data to data from other sources under future IRB approved protocols, patient identifying information will be retained in the archived database.

Initial data collection will include for each individual patient: baseline patient demographics, histology, Karnofsky performance status (6), presence or absence of systemic metastatic disease outside the brain, controlled or uncontrolled primary cancer, number of brain metastases, size and volume of largest brain metastasis treated as well as total volume of brain disease treated, dose prescribed and dose delivered to each brain lesion, and steroid use.

Patients will be followed every three months with history, clinical examination and brain MRI for 1 year. Follow up data collection at each follow-up visit will include the following:

1. Central nervous system toxicity such as Central nervous system necrosis, based on the CTCAE version 4.0.

2. Local failure in the brain on follow-up MRI defined as follows :
   A. At least 20% increase in sum longest distance relative to lesion size prior to radiation treatment (reference 7 describes the RANO group criteria which is slightly different and uses nadir size rather than size prior to radiation)

3. Distant failure in the brain defined as additional new brain metastatic lesion(s) outside the irradiated region.

4. Overall survival.

If new lesion(s) are identified during follow up, the patient will be considered for FFSRT on protocol at the discretion of the treating physician. These new lesions are also followed every 3 months for 1 year with MRI and are considered for local control (LC) at the 6 month time point.

5.0 Treatment Planning Guidelines
All patients treated on this prospective study will be treated with FFRST with consistent setup, contrast-based CT simulation, MRI-fused contouring of tumor and normal tissue structures, and with standard dose prescriptions based on size of tumor and MD Anderson CNS Radiation stereotactic treatment planning guidelines for normal tissue tolerances.

All patients will be simulated supine in a CT-simulator with an aquaplast mask, bite block, and head holder. IV contrast will be required. Image acquisition will be at 1.0-mm slice thickness. A volumetric brain MRI will be obtained to be fused with the CT images obtained for treatment planning. A volumetric gadolinium-enhanced brain MRI is performed with 1.0-mm slice thickness to optimize disease extent, volume and aid in fusion with the CT simulation image set. If a volumetric brain MRI is not available, then comparable high-quality MRI with contrast enhancement obtained within 5 days of the CT simulation may be deemed acceptable for fusion by the treating physician.

The volumetric brain MRI will be fused with the CT simulation image set for optimal tumor localization and the tumor contoured on the CT data set. The gross lesion will be defined as the gross tumor volume (GTV). A 2 mm volumetric expansion will be placed on the GTV to create the planning target volume (PTV).

The following will be the dose prescription to the PTV based on maximum diameter of each individual brain lesion:

1. < 3.0 cm: 27 Gy/3 fractions
2. 3.0 - 3.9 cm: 30 Gy/5 fractions
3. 4.0- 5.0 cm: 25 Gy/5 fractions

Treatment planning will be performed with three-dimensional, intensity-modulated radiation therapy (IMRT) or volumetric modulated arc therapy (VMAT) techniques. Normal tissue (brain stem, optic nerves, optic chiasm, brainstem) constraints of dose are the following:

For **3 fraction** cases brain stem, optic nerves, optic chiasm, and brainstem, maximum point dose should not exceed 16 Gy in patients with no previous brain radiation, and 1 cc should not receive over 12 Gy.

For **5 fraction** cases brain stem, optic nerves, optic chiasm, and brainstem, maximum point dose should not exceed 22 Gy in patients with no previous brain radiation and 1 cc should not receive 20 Gy. Optimally the chiasm and optic nerve point dose should be kept less than 18 Gy and <0.02cc should receive less than 16.5 Gy but for tumors adjacent to the chiasm or optic nerve the former constraints are acceptable.

For all cases, mean brain dose should not exceed 8 Gy.

Treatment delivery will require the following quality assurance measures prior to each fraction of treatment:

1. Presence of radiation physicist, radiation oncologist and radiation therapist at the treatment console.

2. kV orthogonal imaging prior to cone-beam CT (CBCT) to confirm gross alignment of the patient's head and isocenter placement as compared to orthogonal digitally reconstructed radiographs (DRRs) generated from the treatment plan.
3. Non-contrast CBCT with appropriate isocenter shifts directed by the radiation oncologist to confirm match of metastatic target lesion seen on real-time cone-beam CT with planning CT contour of GTV. If the metastatic target lesion is unable to be seen on the non-contrast CBCT, then alignment of normal tissue brain structures near the metastatic target lesion will be necessary instead, such as bony skull and ventricles.
4. MV orthogonal imaging after CBCT and just prior to treatment delivery to confirm isocenter placement as compared to DRRs.

6.0 Statistical Considerations

Patients will be treated based on an optimal plan derived for each patient. We plan to enroll a maximum of 35 patients. We expect to encounter 2 lesions requiring treatment per patient. The primary endpoint is lesion failure within the first 6 months based on imagining assessments for each lesion. The evaluable study population will consist of patients who reach the 6-month evaluation point for local control. We will assume that failure of a lesion is independent of failure for all other lesions. However, an exploratory analysis will be conducted to assess this assumption as evidence suggesting within patient correlation is not available in the published literature.

Patients' lesions will be followed for failure starting on day 1 of radiation treatment in 3-month intervals (+/-2 months) for up to 1-year. Patients unable to complete at least 80% of their prescribed dose will be replaced.

We plan to evaluate efficacy at the level of the lesion. However, this study will monitor futility attributable to patient benefit at the patient-level using local control in consort with the assessment criteria for lesions.

6.1 Efficacy Analysis

Efficacy will be estimated by providing the incidence of lesion failure by 6-months. Assuming we will enroll patients providing 2 lesions on average, we expect to acquire 70 lesions for the entire cohort of 35 treated patients. The information below provides the estimated boundaries of exact 95% CIs for varying sample sizes. As futility monitoring is employed at the patient-level, there is potential to terminate the trial early. We presuppose 40 lesions if the trial is stopped after the first stage, and 70 lesions if the trial runs through both stages. Details regarding the futility assessment are described below.

The sample size justification for our primary endpoint is based on the incidence of lesion failure. By assuming a 6-month incidence rate of 0.50, the bounds of the 95% exact CI will not extend beyond these limits: (0.34, 0.66) with 40 lesions provided by 20 patients. Furthermore, assuming a 6-month incidence rate of 0.50, the bounds on the 95% exact CI will not extend beyond these limits: (0.38, 0.62) with 70 lesions provided by 35 patients.

Following the description of the two-stage design below is a sample size justification based on the precision associated with 95% CIs utilizing assumptions made with patient-level data.

6.2 Futility Monitoring

Futility monitoring will be conducted to stop the trial early if there is no observed benefit at the patient-level. The patient-level LC rate at 6-months will be used to monitor the trial for futility. We will estimate the LC rate at 6 months. The Green-Dahlberg two-stage design (http://www.swogstat.org/stat/public/Help/twostage.html) will be used to terminate the trial early for futility. The null hypothesis is that the 6-month LC rate is ≤ 55% and is not as effective as previously believed, thus requiring early termination of the trial for futility. The alternative hypothesis is that the LC rate at 6-months is clinically advantageous as demonstrated by a 6-month LC rate ≥ 75%. Hypothesis testing will be conducted using a one-sided test, alpha = 0.05, and Type II error of 0.20. The probability of stopping at the end of Stage 1 is 0.409 if the null hypothesis is true, and the probability of stopping at the end of Stage 1 is 0.014 if the alternative hypothesis is true.

We will enroll a total of 35 evaluable patients. Initially, 20 patients will be enrolled in the first Stage of the study and evaluated for response at 6-months from day 1 of radiation treatment. If there are at least 11 patients among the first 20 who achieve LC at 6-months, then an additional 15 patients will be enrolled and evaluated. Note that the 20th patient in the first Stage must be evaluated for response prior to continuing to the second stage when less than 11 patients have experienced LC at 6-months. Finally, if there are at least 25 patients achieving LC at 6-months among all 35 patients, then we will reject H0. The statistical assessments will be used to design and appropriately power a Phase III study if warranted based on the outcomes we observe in this Phase II study.

Figure 1 Study Schema

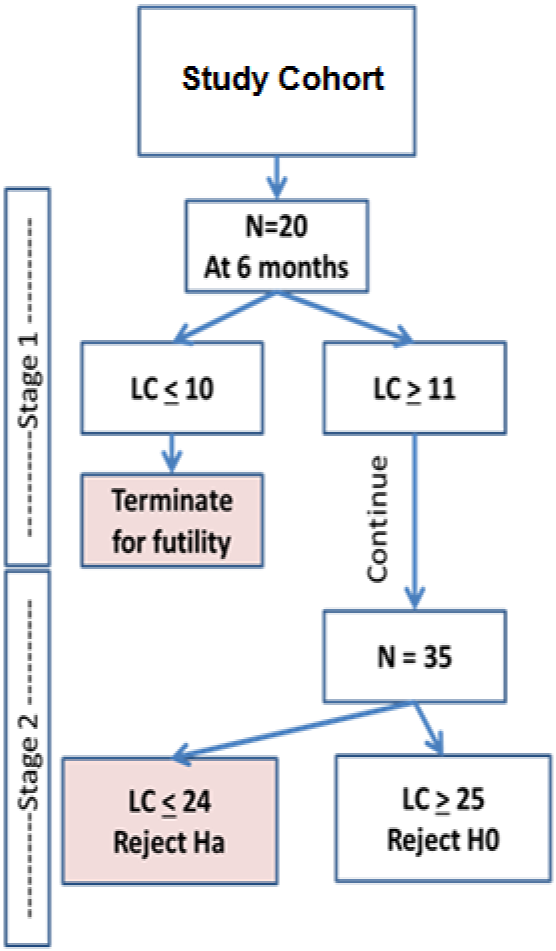

Table 1. Description of the 2 Stage Design

	Study Cohort	
Null Hypothesis	LC at 6 months ≤ 55%	
Alternative Hypothesis	LC at 6 months ≥ 75%	
Sample Size for Stage 1 (n1)	20	
Sample Size for Stage 2 (n2)	15	
Total Sample Size (n)	35	
Terminate Cohort for Futility if this many Success (A1) are observed in Stage 1	10 or fewer out of 20	
Continue to Stage 2 if this many Successes are observed	11 or more out of 20	
# of LCs (R2) required to reject H0 at the end of the trial	25 or more out of 35	

For patient-level data, we will report the estimated LC rate at 6-months along with a 95% exact CI for the study cohort. Assuming a LC rate of 0.50, the bounds of the 95% exact CI will not extend beyond these limits: (0.27, 0.73) with 20 patients. Assuming a LC rate of 0.60, the bounds on the 95% exact CI will not extend beyond these limits: (0.42, 0.76) with 35 patients.

6.3 Analysis of Study Results

Descriptive statistics will be used to explore the data for quality issues, identifying outliers, and providing summaries of data distributions. T-tests or Wilcoxon rank sum tests will be used to compare continuous variables between patient groups of interest. Categorical data will be summarized using frequencies and percentages. Chi-square or Fisher's exact tests will be used to compare categorical variables between patient subgroups of interest. The LC estimates at the patient-level and incidence of failure at the lesion-level will be reported and will be accompanied by a 2-sided exact 95% confidence interval.

Rates of local control will be estimated at each of the follow-up assessments. Intracranial progression-free survival and overall survival will be summarized using the Kaplan-Meier method. Intracranial progression-free survival will be defined as the time from enrollment to either the first observation of progressive disease in the brain or death due to any cause. For intracranial PFS, patients not experiencing a death or intracranial progression will be censored at the last tumor assessment, approximately 1 year from day 1 of the start of radiation treatment. Intracranial PFS and OS will be summarized using the Kaplan-Meier method, and the 50th percentile of the K-M distribution will determine the median intracranial PFS or OS.

Finally, the cost data will be summarized using descriptive statistics such as the mean, median, standard deviation, and range. Comparisons in cost between treatment modalities will be evaluated using a paired t-test where each patient will serve as his or her own control to determine if the cost of FFSRT is comparable to standard treatment.

6.4 Safety Monitoring

Neurotoxicities are expected and manageable; however, we will monitor for unreasonably excessive grade 3 or higher neurotoxicities. To assess safety, data on neurotoxicity will be tabulated by grade for the study cohort. The neurotoxicity burden for the trial will be computed by summing the number of neurotoxicities experienced by a patient, and then dividing by the total number of patients on trial.

Neurotoxicity (NT) monitoring will be conducted independently for each study cohort. We will use the method described by Thall et al. (1995) to sequentially monitor the rate of grade 3 or higher treatment related neurotoxicities starting from day 1 of FFSRT to a patient's first follow-up assessment at 3 months. If we have reason to believe that the grade 3 or higher NT rate is more than 33%, we will terminate the FFSRT cohort. NT monitoring will occur in cohorts of size 5. We will stop the trial early if at any time the

Pr(rate of grade 3 or higher NT at 3 months from day 1 of FFSRT > 33% | data from the trial) > 0.90.

That is, given the outcomes from the patients who have already been evaluated, if we determine there is more than a 90% chance that the grade 3 or higher NT rate is greater than 33%, we will stop the trial. We will assume a beta (1,1) prior distribution for the grade 3 or higher NT rate. Therefore, the trial will be stopped for excessive toxicity if at any time one of the following boundaries is crossed:

[# of patients with grade 3 or higher NT at 3 months from day 1 of FFSRT / # of patients evaluated]

≥ 3/5, 6/10, 8/15, 10/20, 12/25, 14/30, 16/35

The operating characteristics (OCs) of this decision rule are shown in Table X and are based on 1,000 simulations for each scenario. The OCs indicate that the posterior probability of stopping the trial is 51.4% if the true grade 3 or higher NT rate at 3 months from day 1 of FFSRT- given the data, is greater than 40%.

Table X. Operating characteristics for safety monitoring rule.

P(true)	P(stop)	p10	p25	p50	p75	p90	Avg # pts	Avg # toxs

0.05	0.001	35	35	35	35	35	34.97	1.692
0.1	0.009	35	35	35	35	35	34.73	3.523
0.2	0.073	35	35	35	35	35	32.83	6.554
0.3	0.192	5	35	35	35	35	29.805	8.85
0.4	0.514	5	5	30	35	35	22.49	9.07
0.5	0.851	5	5	5	20	35	14.08	7.135

7.0 Procedure to Obtain Informed Consent

If the treatment recommended for a patient with a solitary or oligometastatic brain lesion(s) is single-fraction frame-based Gamma Knife SRS, the patient will be offered referral to the main campus of MD Anderson or MD Anderson at The Woodlands. If the patient is unable or unwilling to be treated at one of those facilities, likely due to distance or the use of an invasive head frame for treatment, then enrollment on this prospective database study will be offered. The treating radiation oncologist will obtain consent for study participation from the patient. Written informed consent will be obtained on all participants.

8.0 References

1. Radiotherapeutic and surgical management for newly diagnosed brain metastasis(es): An American Society for Radiation Oncology evidence-based guideline Tsao, May N. et al. Practical Radiation Oncology , Volume 2 , Issue 3 , 210 – 225, 2014

2. Hypofractionated Stereotactic Radiosurgery for the Treatment of Large Brain Metastases Tan, D.Y. et al. International Journal of Radiation Oncology • Biology • Physics , Volume 90 , Issue 1 , S701 - S702, 2014

3. Fractionated Stereotactic Radiosurgery for the Treatment of High-Risk Brain Metastases Masino, A. et al. International Journal of Radiation Oncology • Biology • Physics , Volume 90 , Issue 1 , S331 - S332, 2014

4. Comparison of Fractionated Stereotactic Radiosurgery and Single-Fraction Stereotactic Radiosurgery for the Treatment of Brain Metastases Savir, G. et al. International Journal of Radiation Oncology • Biology • Physics , Volume 90 , Issue 1 , S319 - S320, 2014

5.Outcomes From First 3 Years of Frameless Stereotactic Radiosurgery in Treating Brain Metastases Taggar, A. et al. International Journal of Radiation Oncology • Biology • Physics , Volume 90 , Issue 1 , S322, 2014

6.Karnofsky Performance Status Revisited: Reliability, Validity, and Guidelines Schag, C. et al. Journal of Clinical Oncology, Volume 2, No. 3, 187-193, 1984

7. Response assessment criteria for brain metastases:proposal from the RANO group. Lin NU et al. Lancet Oncology, Volume 16, e270-78, 2015.

8. Green SJ and Dahlberg S. Planned Versus Attained Design in Phase II Clinical Trials. Stat Med 11: 853-862, 1992

9. Thall PF, Simon RM, Estey EH. Bayesian sequential monitoring designs for single-arm clinical trials with multiple outcomes. Stat Med 14(4):357-379, 1995
